# Risk Factors Associated with Late Failure of Noninvasive Ventilation in Patients with Chronic Obstructive Pulmonary Disease

**DOI:** 10.1155/2020/8885464

**Published:** 2020-10-13

**Authors:** Tao Chen, Linfu Bai, Wenhui Hu, Xiaoli Han, Jun Duan

**Affiliations:** The Department of Respiratory and Critical Care Medicine, The First Affiliated Hospital of Chongqing Medical University, Chongqing, China

## Abstract

**Background:**

Risk factors for noninvasive ventilation (NIV) failure after initial success are not fully clear in patients with acute exacerbation of chronic obstructive pulmonary disease (COPD).

**Methods:**

Patients who received NIV beyond 48 h due to acute exacerbation of COPD were enrolled. However, we excluded those whose pH was higher than 7.35 or PaCO_2_ was less than 45 mmHg which was measured before NIV. Late failure of NIV was defined as patients required intubation or died during NIV after initial success.

**Results:**

We enrolled 291 patients in this study. Of them, 48 (16%) patients experienced late NIV failure (45 received intubation and 3 died during NIV). The median time from initiation of NIV to intubation was 4.8 days (IQR: 3.4–8.1). Compared with the data collected at initiation of NIV, the heart rate, respiratory rate, pH, and PaCO_2_ significantly improved after 1–2 h of NIV both in the NIV success and late failure of NIV groups. Nosocomial pneumonia (odds ratio (OR) = 75, 95% confidence interval (CI): 11–537), heart rate at initiation of NIV (1.04, 1.01–1.06 beat per min), and pH at 1–2 h of NIV (2.06, 1.41–3.00 per decrease of 0.05 from 7.35) were independent risk factors for late failure of NIV. In addition, the Glasgow coma scale (OR = 0.50, 95% CI: 0.34–0.73 per one unit increase) and PaO_2_/FiO_2_ (0.992, 0.986–0.998 per one unit increase) were independent protective factors for late failure of NIV. In addition, patients with late failure of NIV had longer ICU stay (median 9.5 vs. 6.6 days) and higher hospital mortality (92% vs. 3%) compared with those with NIV success.

**Conclusions:**

Nosocomial pneumonia; heart rate at initiation of NIV; and consciousness, acidosis, and oxygenation at 1–2 h of NIV were associated with late failure of NIV in patients with COPD exacerbation. And, late failure of NIV was associated with increased hospital mortality.

## 1. Introduction

Chronic obstructive pulmonary disease (COPD) is the fourth leading cause of death [1]. Acute exacerbations of COPD are responsible for more than 600,000 hospitalizations annually and result in direct costs of more than $20 billion in the United States [2]. Noninvasive ventilation (NIV) as an effective intervention has been used to manage patients with acute exacerbation of COPD for decades. It improves pH, reduces respiratory rate, reduces PaCO_2_, and subsequently reduces intubation rate and mortality [3, 4]. Because of these advantages, use of NIV in patients with acute exacerbation of COPD has continuously increased in recent years [5, 6]. Moreover, current guidelines strongly recommend NIV to be used in patients with acute exacerbation of COPD [7, 8].

In spite of benefits from NIV in patients with acute exacerbation of COPD, late failure of NIV after initial improvement is not rare. It ranges from 8% to 23% [9–12]. The reasons for early failure of NIV (failure occurred at initial 48 h of NIV) have been widely discussed in patients with acute exacerbation of COPD [13–18]. However, only few studies have reported the reasons for late failure of NIV [9, 12, 19]. Because of small sample sizes, these studies only identified poor sleep, delirium, metabolic complications, and functional limitation were associated with late failure of NIV. Thus, we aimed to find other potential risk factors for late failure of NIV in patients with acute exacerbation of COPD.

## 2. Methods

We performed an observational study in a respiratory ICU of a teaching hospital from January 2012 to December 2015. The study protocol was approved by our ethics committee and the institutional review board (the First Affiliated Hospital of Chongqing Medical University). Because of the observational nature, the informed consents were waived.

Patients who were admitted to our ICU for NIV as a first-line intervention because of acute exacerbation of COPD were screened for eligibility. COPD was diagnosed based on the guideline developed by our Respiratory Disease Committee, Chinese Medical Association in 2002 [20]. We enrolled the patients whose pH was less than 7.35 and PaCO_2_ was more than 45 mmHg which were measured before NIV. However, we excluded those whose NIV was terminated because of clinical improvement, requirement of intubation, or death within 48 h of NIV. Late failure of NIV was defined as intubation or death during NIV after initial success [9].

In our department, NIV was managed by attending physicians, respiratory therapists, and nurses as the protocol reported previously [21]. The face mask (ZS-MZA Face Mask; Shanghai Zhongshan Medical Technology Co., Shanghai, China) was the first choice for NIV (BiPAP Vision or Respironics V60). Patients were positioned at 30° to 45° to avoid aspiration, if there were no contraindications to this positioning. Bi-level positive airway pressure (S/T mode) was used for all patients. Expiratory positive airway pressure was initially set at 4 cmH_2_O and titrated according to the flow curve to ensure that expiratory flow reached zero prior to inspiration or diminished ineffective efforts. However, it was limited to less than 12 cmH_2_O. Inspiratory positive airway pressure was set at 8 cmH_2_O and increased by increments of 2 cmH_2_O to obtain a tidal volume of more than 6 mL/kg or to the maximum tolerated level for each patient. The inspiratory positive airway pressure was limited to less than 25 cmH_2_O. The fraction of inspired oxygen was set to maintain SpO_2_ at around 95%. Humidification was provided by a heated humidifier. If humidification was inadequate, intermittent drinking was allowed. If respiratory failure was reversed, disconnection of NIV equipment was performed per hospital protocol [22].

Intubation was performed referencing the criteria as follows (one major criterion or at least two minor criteria), but it was determined at the discretion of the attending physicians [21]. Major criteria were (1) respiratory arrest, (2) loss of consciousness, (3) hemodynamic instability without response to fluids and vasoactive agents, (4) inability to correct dyspnea, (5) development of conditions necessitating intubation to protect the airway or to manage copious tracheal secretions, and (6) PaO_2_/FiO_2_ below 100 mmHg. Minor criteria were (1) respiratory rate more than 35 breaths/min, (2) blood pH less than 7.30, (3) persistent tachycardia, (4) persistent activation of accessory respiratory muscles, and (5) PaO_2_/FiO_2_ below 150 mmHg.

Nosocomial pneumonia was diagnosed by the methods we reported previously [23]. It was suspected if a patient had a radiographic infiltrate that was new or progressive, along with clinical findings suggesting infection, including new onset of fever, purulent sputum, leukocytosis, and decline in oxygenation. In patients with suspected pneumonia, respiratory tract culture was performed. Samples were obtained by coughing, nasotracheal suction, a protected specimen brush, or bronchoalveolar lavage. Nosocomial pneumonia was confirmed by positive culture and clinical presentations.

Data were analyzed by statistical software (SPSS 17.0; SPSS, Chicago, IL, USA) and reported as mean and standard deviation or median and interquartile range when appropriate. Normally distributed continuous variables were analyzed with the independent-sample *t*-test. Abnormally distributed continuous variables were analyzed with the Mann–Whitney *U* test. Categorical variables were analyzed by the chi-square or Fisher's exact test when appropriate. Within groups, a paired-sample *t*-test was used to analyze the data collected at NIV initiation and 1-2 h of NIV. Kaplan–Meier curves were used to analyze the proportions of intubation in patients with late failure of NIV. Independent risk factors for late failure of NIV were identified by multivariate logistic regression analysis. *p* < 0.05 was considered significant.

## 3. Results

**Figure 1:**
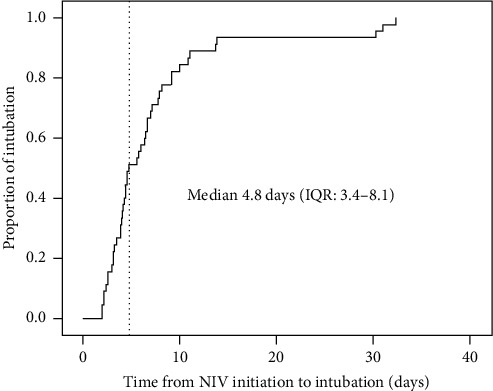
Proportion of intubation in patients with late failure of NIV.

We enrolled 291 patients in this study. After 48 h of NIV, 45 patients experienced intubation. The median time from initiation of NIV to intubation was 4.8 days (interquartile range (IQR): 3.4–8.1) (Figure 1). In addition, 3 patients reached the criteria of intubation. In spite of attending physicians, they did not benefit from intubation and continuous use of NIV. Finally the 3 patients died during NIV. Thus, a total of 48 patients (16%) experienced late NIV failure after initial success.

**Figure 2:**
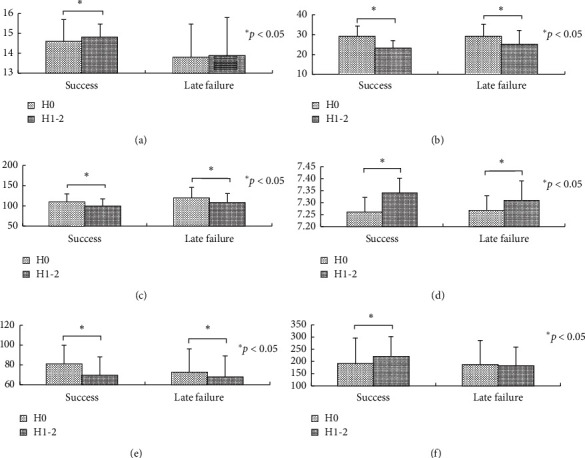
Comparisons between initiation and 1–2 h of NIV: (a) GCS, (b) respiratory rate (breaths/min), (c) heart rate (beats/min), (d) pH, (e) PaCO_2_ (mmHg), and (f) PaO_2_/FiO_2_ (mmHg).

**Figure 3:**
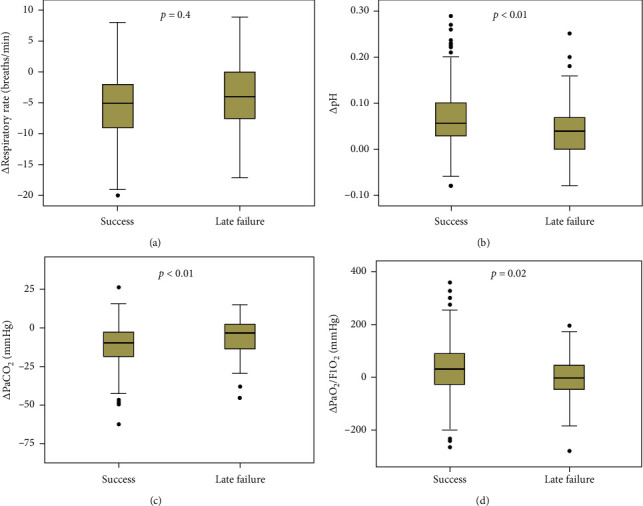
Changes of vital signs from initiation to 1–2 h of NIV.

Patients with NIV success were younger than those with late failure of NIV (71 ± 10 vs. 76 ± 9 years, *p*=0.01) (Table 1). They also had lower APACHE II score (17 ± 6 vs. 21 ± 5, *p* < 0.01) and lower proportion of nosocomial pneumonia (0.8% vs. 14.6%, *p* < 0.01). At initiation of NIV, there were no differences in respiratory rate, pH, and PaO_2_/FiO_2_ between patients with NIV success and late failure of NIV. However, the patients with NIV success had lower respiratory rate (23 ± 4 vs. 25 ± 7 breaths/min, *p*=0.02), higher pH (7.34 ± 0.06 vs. 7.31 ± 0.08, *p*=0.02), and higher PaO_2_/FiO_2_ (221 ± 81 vs. 183 ± 76 mmHg, *p* < 0.01) after 1–2 h of NIV compared with those who experienced late failure of NIV. Compared with the variables collected at NIV initiation, respiratory rate, heart rate, pH, and PaCO_2_ collected at 1–2 h of NIV significantly improved both in NIV success and late failure of NIV groups (Figure 2). However, the respiratory rate, pH, PaCO_2_, and PaO_2_/FiO_2_ improved faster in the NIV success group than those in the late failure of NIV group (Figure 3).

In the multivariate logistic regression analysis, we identified that nosocomial pneumonia (odds ratio (OR) = 75, 95% confidence interval (CI): 11–537), heart rate at initiation of NIV (1.04, 1.01–1.06 beat per min), and pH at 1–2 h of NIV (2.06, 1.41–3.00 per decrease of 0.05 from 7.35) were independent risk factors for late failure of NIV (Table 2). We also found that the Glasgow coma scale (OR = 0.50, 95% CI: 0.34–0.73 per one unit increase) and PaO_2_/FiO_2_ (0.992, 0.986–0.998 per one unit increase) were independent protective factors for late failure of NIV.

Outcomes between patients with NIV success and late failure are summarized in Table 3. There were no differences in duration of NIV and the length of stay in the hospital between the two groups. However, the patients with NIV success had shorter length of stay in the ICU (median 6.6, IQR: 4.9–9.8 vs. 9.5, 5.7–13.8, *p*=0.02) and lower hospital mortality (3% vs. 92%, *p* < 0.01) than those with late failure of NIV.

## 4. Discussion

The current study found the incidence of late failure of NIV was 16% in patients with acute exacerbation of COPD with a relatively large sample size. Although some clinical variables improved both in the NIV success and late failure of NIV groups, the variables in the NIV success group improved faster than those in the late failure of NIV group. Nosocomial pneumonia; heart rate at initiation of NIV; and consciousness, acidosis, and oxygenation at 1–2 h of NIV were associated with late failure of NIV. In addition, late failure of NIV was associated with increased hospital mortality.

The mortality in patients with late failure of NIV was 68% in Moretti's study and 80% in Carratu's study [9, 12]. In our study, the mortality was 92%, which was higher than the value reported by previous studies. We noted that most of the patients experienced NIV failure within 15 days of NIV in our study. However, some cases experienced NIV failure beyond 30 days. From 15 to 30 days of NIV, there was no NIV failure. It indicates that some patients had significantly impaired respiratory function and required prolonged noninvasive ventilation. In addition, longer exposure in the ICU is associated with a higher incidence of nosocomial pneumonia. These reasons contribute much to hospital mortality.

Previous studies reported that patients with late failure of NIV had higher APACHE II score, higher heart rate, lower GCS, and lower blood pressure compared with successful ones [12, 19, 24]. Our study also found similar results. Different from previous studies, we found nosocomial pneumonia was an independent risk factor for late failure of NIV. It reminds us that nosocomial pneumonia played an important role in late failure of NIV. Among the NIV patients who experienced nosocomial pneumonia in our study, NIV failure occurred in 78% of cases. Thus, prevention of nosocomial pneumonia in NIV patients was as important as in those who received invasive mechanical ventilation.

Both in the NIV success and late failure of NIV groups, most of the clinical variables significantly improved after 1–2 h of NIV. However, the respiratory rate, pH, PaCO_2_, and PaO_2_/FiO_2_ improved faster in the NIV success group than those in the late failure of NIV group. These results are new findings compared with previous studies [9, 12, 19, 24]. These data indicate that the patients in the late failure of NIV group responded not so well than those who experienced NIV success. That may be the reason for initial improvement but later failure in the late failure of NIV group.

Our study has several limitations. We found nosocomial pneumonia was associated with late failure of NIV in a patient with acute exacerbation of COPD. However, we only enrolled 9 patients with nosocomial pneumonia. The small sample size may skew this result. Thus, the result is required to be validated with a larger sample size. Secondly, this study was only performed in a respiratory ICU. The single-center study also limited the results to extrapolate to other centers. Thirdly, patients who received intubation later were associated with higher mortality [12, 25]. Therefore, early intubation (e.g., 24 h of NIV) is an alternative to reduce mortality.

## 5. Conclusions

Nosocomial pneumonia; heart rate at initiation of NIV; and consciousness, acidosis, and oxygenation at 1–2 h of NIV were associated with late failure of NIV in patients with COPD exacerbation. In addition, late failure of NIV was associated with increased hospital mortality.

## Figures and Tables

**Table 1:** Baseline characteristics of patients who had NIV success or late failure of NIV.

	NIV success	Late failure of NIV	*p*
	*N* = 243 (84%)	*N* = 48 (16%)	
Age, years	71 ± 10	76 ± 9	0.01^*∗*^
Male/female	186/57	33/15	0.27
APACHE II score	17 ± 6	21 ± 5	<0.01^*∗*^
Nosocomial pneumonia during NIV	2 (0.8%)	7 (14.6%)	<0.01^*∗*^
Data collected at NIV initiation			
GCS	14.6 ± 1.1	13.8 ± 1.7	<0.01^*∗*^
Respiratory rate, breaths/min	29 ± 5	29 ± 6	0.85
Heart rate, beats/min	110 ± 19	120 ± 26	<0.01^*∗*^
MAP, mmHg	102 ± 17	94 ± 18	0.01^*∗*^
pH	7.26 ± 0.06	7.27 ± 0.06	0.80
PaCO_2_, mmHg	81 ± 19	73 ± 23	0.01^*∗*^
PaO_2_/FiO_2_, mmHg	192 ± 103	188 ± 99	0.79
Data collected at 1–2 h of NIV			
GCS	14.8 ± 0.7	13.9 ± 1.9	<0.01^*∗*^
Respiratory rate, breaths/min	23 ± 4	25 ± 7	0.02^*∗*^
Heart rate, beats/min	100 ± 18	109 ± 22	<0.01^*∗*^
MAP, mmHg	91 ± 14	90 ± 16	0.50
pH	7.34 ± 0.06	7.31 ± 0.08	0.02^*∗*^
PaCO_2_, mmHg	70 ± 18	68 ± 21	0.33
PaO_2_/FiO_2_, mmHg	221 ± 81	183 ± 76	<0.01^*∗*^

NIV = noninvasive ventilation; GCS = Glasgow coma scale; MAP = mean arterial pressure. ^*∗*^*p* < 0.05 for NIV success vs. late failure of NIV.

**Table 2:** Univariate and multivariate analysis of risk factors associated with late failure of NIV.

	Univariate analysis		Multivariate analysis	
	OR (95% CI)	*p*	OR (95% CI)	*p*
Age, years	1.05 (1.01–1.08)	<0.01	—	—
APACHE II score	1.11 (1.05–1.18)	<0.01	—	—
Nosocomial pneumonia during NIV	21 (4–103)	<0.01	75 (11–537)	<0.01
Data collected at NIV initiation				
GCS	0.70 (0.57–0.86)	<0.01	—	—
Heart rate, beats/min	1.02 (1.01–1.04)	<0.01	1.04 (1.01–1.06)	<0.01
MAP, mmHg	0.98 (0.96–0.99)	<0.01	—	—
PaCO_2_, mmHg	0.98 (0.97–1.00)	0.01	—	—
Data collected at 1–2 h of NIV				
GCS	0.50 (0.36–0.70)	<0.01	0.50 (0.34–0.73)	<0.01
Respiratory rate, breaths/min	1.08 (1.01–1.14)	0.02	—	—
Heart rate, beats/min	1.03 (1.01–1.04)	0.01	—	—
pH at 1–2 h of NIV, per decrease of 0.05 from 7.35	1.67 (1.26–2.25)	<0.01	2.06 (1.41–3.00)	<0.01
PaO_2_/FiO_2_, mmHg	0.993 (0.988–0.998)	<0.01	0.992 (0.986–0.998)	0.01

OR = odds ratio; CI = confidence interval; NIV = noninvasive ventilation; GCS = Glasgow coma scale; MAP = mean arterial pressure.

**Table 3:** Outcomes between patients with NIV success and late failure.

	NIV success, *N* = 243 (84%)	Late failure of NIV, *N* = 48 (16%)	*p*
Duration of NIV (median (IQR)), days	5.0 (3.5–7.7)	5.2 (3.4–9.0)	0.52
Duration of ICU stay (median (IQR)), days	6.6 (4.9–9.8)	9.5 (5.7–13.8)	0.02^*∗*^
Duration of hospital stay (median (IQR)), days	13.0 (8.3–19.1)	14.1 (9.7–22.7)	0.41
Hospital mortality	8 (3%)	44 (92%)	<0.01^*∗*^

NIV = noninvasive ventilation; IQR = interquartile range; ICU = intensive care unit. ^*∗*^*p* < 0.05 for NIV success vs. late failure of NIV.

## Data Availability

The datasets analyzed during this study are available from the corresponding author upon reasonable request.

## Abbreviations

COPD: Chronic obstructive pulmonary disease
NIV: Noninvasive ventilation
GCS: Glasgow coma scale
MAP: Mean arterial pressure
OR: Odds ratio
CI: Confidence interval
IQR: Interquartile range
ICU: Intensive care unit.

## References

[1] Vestbo J., Hurd S. S., Agustí A. G. (2013). Global strategy for the diagnosis, management, and prevention of chronic obstructive pulmonary disease. *American Journal of Respiratory and Critical Care Medicine*.

[2] Snow V., Lascher S., Mottur-Pilson C. (2001). Evidence base for management of acute exacerbations of chronic obstructive pulmonary disease. *Annals of Internal Medicine*.

[3] Lightowler J. V., Wedzicha J. A., Elliott M. W. (2003). Non-invasive positive pressure ventilation to treat respiratory failure resulting from exacerbations of chronic obstructive pulmonary disease: cochrane systematic review and meta-analysis. *British Medical Journal*.

[4] Ram F. S. F., Picot J., Lightowler J., Wedzicha J. A. (2004). Non-invasive positive pressure ventilation for treatment of respiratory failure due to exacerbations of chronic obstructive pulmonary disease. *The Cochrane Database of Systematic Reviews*.

[5] Chandra D., Stamm J. A., Taylor B. (2012). Outcomes of noninvasive ventilation for acute exacerbations of chronic obstructive pulmonary disease in the United States, 1998–2008. *American Journal of Respiratory and Critical Care Medicine*.

[6] Mihaela M. S., Shieh M.-S., Pekow P. S., Hill N., Rothberg M. B., Lindenauer P. K. (2015). Trends in mechanical ventilation among patients hospitalized with acute exacerbations of COPD in the United States, 2001 to 2011. *Chest*.

[7] Hill W., Qing-yuan Z. (2007). Guideline for mechanical ventilation in patients with acute exacerbation of chronic obstructive pulmonary disease (2007). *Zhongguo Wei Zhong Bing Ji Jiu Yi Xue*.

[8] Keenan S. P., Sinuff T., Burns K. E. A. (2011). Clinical practice guidelines for the use of noninvasive positive-pressure ventilation and noninvasive continuous positive airway pressure in the acute care setting. *Canadian Medical Association Journal*.

[9] Carratu P., Bonfitto P., Dragonieri S. (2005). Early and late failure of noninvasive ventilation in chronic obstructive pulmonary disease with acute exacerbation. *European Journal of Clinical Investigation*.

[10] Carrillo A., Ferrer M., Gonzalez-Diaz G. (2012). Noninvasive ventilation in acute hypercapnic respiratory failure caused by obesity hypoventilation syndrome and chronic obstructive pulmonary disease. *American Journal of Respiratory and Critical Care Medicine*.

[11] Brochard L., Mancebo J., Wysocki M. (1995). Noninvasive ventilation for acute exacerbations of chronic obstructive pulmonary disease. *New England Journal of Medicine*.

[12] Moretti M., Cilione C., Tampieri A. (2000). Incidence and causes of non-invasive mechanical ventilation failure after initial success. *Thorax*.

[13] Ozyilmaz E., Ugurlu A. O., Nava S. (2014). Timing of noninvasive ventilation failure: causes, risk factors, and potential remedies. *BMC Pulmonary Medicine*.

[14] Confalonieri M., Garuti G., Cattaruzza M. S. (2005). A chart of failure risk for noninvasive ventilation in patients with COPD exacerbation. *European Respiratory Journal*.

[15] Carlucci A., Richard J.-C., Wysocki M., Lepage E., Brochard L. (2001). Noninvasive versus conventional mechanical ventilation. *American Journal of Respiratory and Critical Care Medicine*.

[16] Miller D., Fraser K., Murray I., Thain G., Currie G. P. (2012). Predicting survival following non-invasive ventilation for hypercapnic exacerbations of chronic obstructive pulmonary disease. *International Journal of Clinical Practice*.

[17] Phua J., Kong K., Lee K. H., Shen L., Lim T. K. (2005). Noninvasive ventilation in hypercapnic acute respiratory failure due to chronic obstructive pulmonary disease vs. other conditions: effectiveness and predictors of failure. *Intensive Care Medicine*.

[18] Chakrabarti B., Angus R. M., Agarwal S., Lane S., Calverley P. M. A. (2009). Hyperglycaemia as a predictor of outcome during non-invasive ventilation in decompensated COPD. *Thorax*.

[19] Campo F. R., Drouot X., Thille A. W. (2010). Poor sleep quality is associated with late noninvasive ventilation failure in patients with acute hypercapnic respiratory failure. *Critical Care Medicine*.

[20] Chinese Medical Association Respiratory Disease Committee (2002). Guideline for the diagnosis and treatment of chronic obstructive pulmonary disease. *Chinese Journal of Tuberculosis and Respiratory Disease*.

[21] Fan L., Zhao Q., Liu Y., Zhou L., Duan J. (2014). Semiquantitative cough strength score and associated outcomes in noninvasive positive pressure ventilation patients with acute exacerbation of chronic obstructive pulmonary disease. *Respiratory Medicine*.

[22] Duan J., Tang X., Huang S., Jia J., Guo S. (2012). Protocol-directed versus physician-directed weaning from noninvasive ventilation. *The Journal of Trauma and Acute Care Surgery*.

[23] Zhang Z., Duan J. (2015). Nosocomial pneumonia in non-invasive ventilation patients: incidence, characteristics, and outcomes. *Journal of Hospital Infection*.

[24] Ciledag A., Kaya A., Ercen Diken O. (2014). The risk factors for late failure of non-invasive mechanical ventilation in acute hypercapnic respiratory failure. *Tuberk Toraks*.

[25] Esteban A., Frutos-Vivar F., Ferguson N. D. (2004). Noninvasive positive-pressure ventilation for respiratory failure after extubation. *New England Journal of Medicine*.

